# Assessing the Effects of Water Deficit on Photosynthesis Using Parameters Derived from Measurements of Leaf Gas Exchange and of Chlorophyll *a* Fluorescence

**DOI:** 10.3389/fpls.2017.02068

**Published:** 2017-12-14

**Authors:** Laurent Urban, Jawad Aarrouf, Luc P. R. Bidel

**Affiliations:** ^1^UMR 95 Qualisud/Laboratoire de Physiologie des Fruits et Légumes, Université d'Avignon, Avignon, France; ^2^INRA, UMR 1334 AGAP, Montpellier, France

**Keywords:** water deficit, photosynthesis, chlorophyll *a* fluorescence, leaf gas exchange, stomatal conductance, tolerance mechanisms, induction curves of maximal chlorophyll fluorescence

## Abstract

Water deficit (WD) is expected to increase in intensity, frequency and duration in many parts of the world as a consequence of global change, with potential negative effects on plant gas exchange and growth. We review here the parameters that can be derived from measurements made on leaves, in the field, and that can be used to assess the effects of WD on the components of plant photosynthetic rate, including stomatal conductance, mesophyll conductance, photosynthetic capacity, light absorbance, and efficiency of absorbed light conversion into photosynthetic electron transport. We also review some of the parameters related to dissipation of excess energy and to rerouting of electron fluxes. Our focus is mainly on the techniques of gas exchange measurements and of measurements of chlorophyll *a* fluorescence (ChlF), either alone or combined. But we put also emphasis on some of the parameters derived from analysis of the induction phase of maximal ChlF, notably because they could be used to assess damage to photosystem II. Eventually we briefly present the non-destructive methods based on the ChlF excitation ratio method which can be used to evaluate non-destructively leaf contents in anthocyanins and flavonols.

## Introduction

Water deficit (WD) is expected to increase in intensity, frequency and duration in many parts of the world, notably in Africa, Asia and Central and South America, as a consequence of climate change (IPCC, 2014). WD is generally perceived as negative for plants basically because it can lead to stress which may in turn threaten plant survival. More commonly, WD impairs plants' photosynthetic rate and growth, thus potentially disturbing balances existing between species competing in natural habitats (Smith and Huston, 1990; Nambiar and Sands, 1993) while reducing plant productivity in cropping systems (Boyer, 1982). The latter issue has received much attention because decreases in crop productivity challenge food security (Hanjra and Qureshi, 2010). Besides, reduced production of photosynthetic products may also impair osmotic adjustment and the capacity of plants to cope with drought (Blum, 2017). Dealing with the negative effects of WD on growth and productivity will require, among others, being able to assess the way WD impacts photosynthesis, and to interpret plants' responses correctly within integrated views of their strategies. Of course, the issue of the impact of WD on growth and productivity is a complex one that cannot be reduced to a simple negative effect on photosynthesis, since WD may impact also developmental processes. The latter, not only the former, are involved in productivity (e.g., flowering and fruiting). Despite these limitations, leaf photosynthesis analysis remains pivotal in all WD studies. Moreover, it is quite clear that plants experience multiple stress situations in natural or field conditions, and that their responses to a combination of stresses cannot be extrapolated simply from separate studies of individual stresses (Mittler, 2006). In the case of WD there is at least the need to take into account the light conditions. Eventually, it is important not to forget that in addition to net photosynthetic CO_2_ assimilation per unit area and time (A_net_), leaf area and distribution, as well as mitochondrial respiration are also important for growth and production. Mitochondrial respiration may not only contribute to significant carbon losses, especially under stress conditions, reducing the net carbon gain (Van Oijen et al., 2010; Sperlich et al., 2015), it is also a key regulator of the energy status of plants under stress.

**Figure 1:**
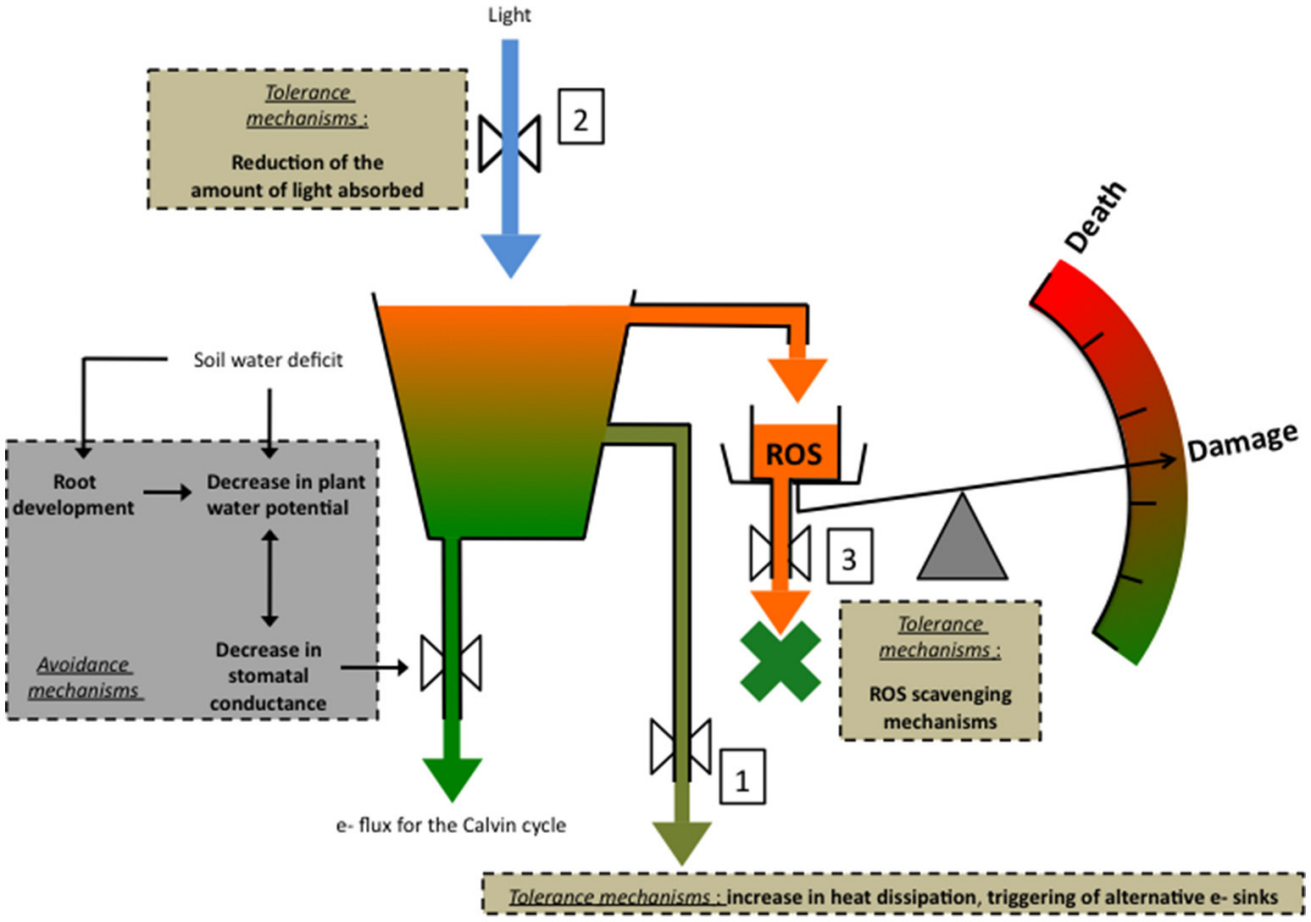
A simplified representation of the major tolerance mechanisms against drought-associated photooxidative stress in conditions of high light. Avoidance mechanisms are also represented: (1) decreasing plant water potential (Ψ) improves plant capacity to remove water from the drying soil, whereas (2) decreasing stomatal conductance (g_s_), exerts a positive effect on the plant water content by saving water. (3) root development increases plant capacity to take up water and therefore to maintain a high water content. In high light conditions, the decrease in g_s_ entails a decrease in the quantity of CO_2_ entering the leaf, therefore creating an imbalance between the energy capture and energy use by photochemistry. The risk for excess energy to form potentially damaging reactive oxygen species (ROS) increases and must be mitigated by energy dissipation processes and the triggering of alternative e- sinks 1, and by processes aiming at decreasing the quantity of light entering the leaf 2. If these mechanisms fail to prevent ROS formation, the latter can be eliminated by ROS scavenging processes 3. In the case these mechanisms are insufficient, ROS can damage notably ATP synthase, leading to a decrease in RuBP synthesis and Rubisco activity. Eventually damage may lead to death.

A_net_ is determined by stomatal conductance (g_s_) and mesophyll conductance (g_m_), which determine CO_2_ supply to carboxylation sites, and also by the photosynthetic metabolic potential (A_pot_), which determines the capacity of the photosynthetic machinery to process CO_2_. A_pot_ depends on the amount and activities of the components of the light-harvesting, the electron transport and the energy-transduction processes, as well as by the carbon metabolism components, including such enzymes as the Rubisco and processes like RUBP synthesis by the Calvin cycle (Lawlor and Cornic, 2002; Flexas et al., 2004; Chaves et al., 2009; Lawlor and Tezara, 2009). Mild WD decreases A_net_ via a reduction in g_s_. In low light conditions, photosynthetic activity, notably electron transport and NADP^+^ reduction are maintained. But in high light conditions, since A_net_ does not increase, an imbalance between energy capture and energy use by photochemistry occurs, leading to a decrease in the rate of linear electron transport, downregulation of ATP synthase activity, which allows to keep a high level of ΔpH and of energy dissipation (Kanazawa and Kramer, 2002), and the triggering of alternative electron routes. These mechanisms may not be efficient enough to prevent the formation of reactive oxygen species (ROS) whereas scavenging mechanisms may be overflown to the point of allowing accumulation of ROS. Lawlor and Tezara (2009) hypothesized that the latter damage ATP synthase, leading to a decrease in ATP and consequently in RuBP synthesis by the Calvin cycle, and eventually Rubisco activity. In case of severe stress, damage can even lead to death (Figure 1).

This paper has not the ambition to provide a full and detailed review of the consequences of drought on photosynthesis (see Lawlor and Tezara, 2009; Pinheiro and Chaves, 2011 for instance) and on growth (Farooq et al., 2009), but to provide a review of those parameters related to photosynthesis that can be derived from measurements of gas exchange and chlorophyll *a* fluorescence (ChlF) that are performed on leaves, in the field. Recently, a new generation of fluorimeters was made available that provide the high time resolution needed for performing measurements of fast ChlF induction kinetics. Parameters derived from analysis of the so-called OJIP transients are used to analyze the response of PSII to stress, but some of them may also be used as indicators of energy use efficiency, photoinhibition and even damage (Ripoll et al., 2016b). We shall put some emphasis on them in this review. Marginally we shall invoke also a few parameters of remote sensing which could be used in complement or as substitutes.

For readers not familiar with ChlF measurements, there are three major classes of instruments. The first class encompasses devices based on the concept of a single turnover flash (STF), the second class of instruments exploits a saturating pulse for analysis of the induction curve of maximal ChlF (i.e., the analysis of so-called OJIP transients) and the last one is designed to study steady state fluorescence for quenching analysis and for coupled ChlF and gas exchange measurements (Kalaji et al., 2014). In the first class, STF devices provide among other things information on the electron transfer reactions within PSII. Although potentially useful to characterize responses to stress, they are not commonly used in field studies and will therefore not be included in this review. For the same reason we excluded thermoluminescence (a delayed fluorescence that gives information on the occurrence of recombination reactions in PSII as a function of the redox state of the electron transport chain), as well as 77 K fluorescence and fast and ultra-fast fluorescence. For the reader interested in these techniques we suggest the following articles and reviews: Shinkarev (2005) for STF, Misra et al. (2001) and Ducruet and Vass (2009) for thermoluminescence, Goltsev et al. (2009) for delayed fluorescence, Srivastava and Strasser (1999) and Papageorgiou (2011) for 77 K fluorescence, and Holzwarth (2008) and Berera et al. (2009) for fast fluorescence techniques. The second class of instruments makes use of strong light pulses of few 100 ms, to obtain information on the photosynthetic electron transport chain (ETC), its reduction kinetics, Photosystem II (PSII) antenna size and relative content of ETC components. The instruments of the last class are designed to measure ChlF intensity in the steady state, as affected by the redox state of the ETC and by changes in the ChlF yield. The analysis of the causes for yield changes is called quenching analysis. Modulated light is used as a trick to separate the effect of actinic light that drives photosynthesis and the low-intensity measuring light that is used to probe the state of the photosynthetic system on the measured ChlF intensity (Kalaji et al., 2014). Besides quenching analysis, pulsed amplitude modulated fluorimeters can be used in combination with gas exchange measurement systems to study the interactions between the ETC, the Calvin-Benson cycle, CO_2_ conductance and photorespiration. It is not our objective here to provide the reader with the theoretical background, the assumptions behind the models, and practical considerations of all the techniques evoked in this review. Below is a very small selection of papers and books among many readers who intend to familiarize themselves with these techniques may find useful:

- for gas exchange measurements (von Caemmerer and Farquhar, 1981; Nobel, 2009);
- for OJIP transient measurements, performed on dark-adapted leaves (Stirbet, 2011; Kalaji et al., 2014; Goltsev et al., 2016).
- for steady state fluorescence measurements under modulated light (Maxwell and Johnson, 2000; Logan et al., 2007; Murchie and Lawson, 2013; Kalaji et al., 2014).

We shall now put in perspective the parameters derived notably from measurements of leaf gas exchange and ChlF, by considering successively g_s_, g_m_, the components of photosynthetic capacity, light absorbance, efficiency of absorbed light conversion into photosynthetic electron transport, rerouting of electron fluxes and dissipation of excess energy. We shall then present the ChlF techniques that can be used to assess leaf concentrations in anthocyanins and flavonols, which may play a role as antioxidants, and eventually review the parameters that could be used to analyze photodamage. The symbols used in this review are listed in Tables 1, 2. Specific portable field measurement systems are mentioned but we have not the ambition here to provide an exhaustive list.

**Table 1:** List of symbols.

A_gross_, A_net_	Gross and net photosynthetic rate
A_max_	Maximal rate of net photosynthesi
A_pot_	Photosynthetic metabolic potential
ATP	Adenosine triphosphate
CET	Cyclic electron transport
ChlF	Chlorophyll fluorescence
C_c_	CO_2_ concentration at the carboxylation site
C_i_	Intercellular CO_2_ concentration
CWSI	Crop water stress index
ETC	Electron transport chain
${\text{F}}_{\text{o}}^{\prime }$, ${\text{F}}_{\text{m}}^{\prime }$	Minimal and maximal values of ChlF of light-adapted leaves
g_m_	Mesophyll conductance
g_s_	Stomatal conductance
J, J_T_, ETR	Electron transport rate
J_A_	Electron transport rate for alternative sinks
J_C_	Electron transport rate for carboxylation
J_O_	Electron transport rate for oxygenation
J_max_	Light-saturated electron transport rate
LUE	Light use efficiency
N_a_	Leaf nitrogen content per unit leaf area
N_m_	Leaf nitrogen content expressed on leaf mass basis
NADPH	Nicotinamide adenosine diphosphate (reduced)
NDH	NADH dehydrogenase-like
NPQ	Non photochemical quenching
OEC	Oxygen evolving complex
PC, PQ	Plastocyanins, Plastoquinones
PRI	Photochemical Reflectance Index
PSII	Photosystem II
Q	Photosynthetically active flux density
R_d_	Rate of mitochondrial respiration in the presence of light
R_n_	Rate of mitochondrial respiration in the absence of light
ROS	Reactive oxygen species
Rubisco	Ribulose 1,5-diphosphate carboxylase, oxygenase
RuBP	Ribulose 1,5-diphosphate
SPS	Sucrose phosphate synthase
STF	Single turnover flash
T_a_	Air temperature
T_c_	Canopy temperature
TPU	Triose-phosphate utilization
Y_NO_	Quantum yield of non-light induced NPQ of ChlF
Y_NPQ_	Quantum yield of light induced NPQ of ChlF
V_cmax_	Maximal carboxylation rate of Rubisco
WD	Water deficit
α	Initial quantum efficiency of PSII
Φ_PSII_	Efficiency of absorbed light conversion
θ	Leaf absorbance
Γ	Light compensation point
Γ^*^	CO_2_ compensation point
τ	Specificity factor of Rubisco

*The symbols for the so-called OJIP parameters are presented in Table 2*.

**Table 2:** Definition of some common OJIP/OKJIP parameters (after Strasser et co-workers), including F_0_, F_m_, F_v_, and F_v_ /F_m_.

**Parameter**	**Definition**
F_0_	Initial value of ChlF, generally taken at 20 or 50 μs (O-level)
F_k_	ChlF value at 300 μs (K-level)
F_j_	ChlF value at 2 ms (J-level)
F_i_	ChlF value at 30 ms (I-level)
F_m_	Maximum value of ChlF under saturating light (P-level)
F_v_ = F_m_ - F_0_	Maximum variable ChlF
F_v_/F_m_	Maximum quantum yield of primary PSII chemistry
V_k_ = (F_k_ - F_0_)/F_v_	Relative variable ChlF at 300 μs
V_j_ = (F_j_ - F_0_)/F_v_	Relative variable ChlF at 2 ms
V_i_ = (F_i_ - F_0_)/F_v_	Relative variable ChlF at 30 ms
M_0_ = 4 ms^−1^.V_k_	Initial slope of relative variable ChlF for F_0_ taken at 50 μs
Area	Area between the OJIP/OKJIP curve and the F_m_ line
S_m_ = Area/F_v_	Normalized area
N = S_m_/(M_0_/V_j_)	Turnover number
J^ABS^ = J^TR^ + J^DI^	Rate of photon absorption by PSII antenna (absorbed photon flux)
${\text{J}}_{0}^{\text{TR}}$	Maximum, initial rate of exciton trapping by all PSII reaction centers (maximum trapped exciton flux)
J^DI^	Rate of energy dissipation in PSIIs by processes other than trapping (dissipated energy flux)
${\text{J}}_{0}^{\text{ET}2}$	Electron transport flux from protein protein Q_A_ to protein Q_B_
${\text{J}}_{0}^{\text{RE}1}$	Electron transport flux until PSI acceptors (at 30 ms)
J^ABS^/RC = (M_0_/V_j_)/(F_v_/F_m_)	Average absorbed photon flux per PSII reaction centers/apparent antenna size of an active PSII
${\text{J}}_{0}^{\text{TR}}$/RC = M_0_/V_j_	Maximum trapped exciton flux per PSII
J^DI^/RC = J^ABS^/RC − ${\text{J}}_{0}^{\text{TR}}$/RC	Dissipated energy flux per PSII
PI_ABS_ = (RC/J^ABS^).(F_v_/F_0_).(1 − V_j_)/V_j_	Performance index for energy conservation from photons absorbed by PSII antenna to the reduction of protein Q_B_
RC/J^ABS^	Contribution to the PI of the density of active reaction (in the sense of Q_A_ reducing) centers on a chlorophyll basis
F_v_/F_0_	Contribution to the PI of the light reactions for primary photochemistry, i.e. the performance due to the trapping probability
(1 - V_j_)/V_j_	Contribution to the PI of the dark reactions, or, in other words, the performance due to the conversion of excitation energy to photosynthetic electron transport
${\text{PI}}_{\text{ABS}}^{\text{TOT}}$ = PI_ABS_.(1 – V_i_)/(V_i_- V_i_)	Performance index for energy conservation from photons absorbed by PSII antenna until the reduction of PSI acceptors

## Stomatal conductance (g_s_)

Whereas decreasing plant water potential and stimulating root development both result in increased water uptake, stomatal closure results in improved plant water balance and water status by acting on the other end of the water flux chain, namely by limiting transpiration losses. Stomatal functioning has been extensively studied (Damour et al., 2010) and it emerges that g_s_ is arguably the most relevant among all indicators of WD and even plant stress in general. It is certainly one of the first parameters to be affected by WD. Plants can close stomata within minutes upon exposure to WD, thus very efficiently preventing excessive water loss that could endanger them. Stomata represent the major point of control of water fluxes in the so-called soil-plant-atmosphere continuum. Stomatal resistance to water vapor diffusion is indeed the major resistance along the pathway of water from the soil to the atmosphere. Unfortunately stomatal closure may come at a price, which is a limitation to CO_2_ uptake into chloroplasts, a decrease therefore in photosynthesis and growth, and consequently also an increase in the risk of photo-oxidative stress, i.e., the production of potentially damaging and sometimes lethal ROS. It is true that a small decrease in g_s_ impacts transpiration more than photosynthesis (Nobel, 1999) but, in case of more severe drought or in conditions of high light, photosynthesis is inevitably reduced while the risk of photo-oxidative stress increases. To complete the complex picture of stomatal functioning and roles, one must be reminded that stomatal closure, by helping to maintain plant water status, mitigates the drought-associated decrease in plant water potential and therefore the capacity of plants to extract water from a dehydrating soil. It is easy to understand that the ambivalent and pivotal roles of stomata explain why stomatal functioning is such a highly integrated and regulated process in plants (Damour et al., 2010).

Leaf g_s_ is commonly measured in the field using portable gas exchange measurement systems (Table 3). The latter are designed for concomitant measurements of net exchange of CO_2_ in a large range of photosynthetically active flux density (Q), CO_2_ concentration of the air, temperature and humidity. Portable gas exchange measurement systems include the CIRAS-3 (PP systems, Amesbury, USA), the GFS-3000 (Walz Gmbh, Effeltrich, Germany), the LI-6400 and LI-6800 (LI-COR®, Lincoln, USA) and the iFL (Opti-sciences, Hudson, USA).

**Table 3:** Brief overview of the major types of portable devices commonly used for field measurements of photosynthesis-related parameters.

**Type of instrument**	**Nature of measurements**	**Typical parameters**
Portable leaf gas exchange measurement systems	Steady state gas exchanges under controlled conditions A-C_i_ response curves A-Q curves	A_net_, A_max_, transpiration (measured ≪ directly ≫) g_s_, C_i_ (calculated) R_d_ (light off + correction) V_cmax_, J_max_, TPU, Γ^*^ α, Γ
Modulated fluorimeter	ChlF	F_v_/F_m_, F_o_ (on dark-adapted leaves) ${\text{F}}_{\text{v}}^{\prime }$/${\text{F}}_{\text{m}}^{\prime }$, Φ_PSII_ (on light-adapted leaves) NPQ, qP (quenching analysis) J_max_, α (Φ_PSII_-Q curves)
Coupled leaf gas exchange and modulated ChlF measurement systems	Steady state gas exchanges under controlled conditions + ChlF	In addition to all the above-mentioned parameters: g_m_, photorespiration and alternative routes for e- flow
Modulated fluorimeter + dual wavelengths absorbance spectrometer	ChlF + P700 absorption	Cyclic electron transport activity in addition to the usual parameters
Non modulated, high time resolution fluorimeter	Fast ChlF induction kinetics	F_v_/F_m_, F_o_ So-called OJIP parameters (Table 2)
Modulated fluorimeter based on the excitation ratio method	ChlF at different excitation wavelengths	[anthocyanins], [flavonols]
Chlorophyll meter	Leaf transmittance	θ

Leaf (or canopy) temperature can be measured as an alternative to stomatal conductance as an indicator of WD (Jackson et al., 1981). The idea is that when stomata close, the cooling effect associated with transpiration is reduced, resulting in an increase in leaf or canopy temperature. Leaf or canopy surface temperatures can be measured easily through infrared thermography. The measured temperatures can then be exploited to calculate parameters such as the Leaf Temperature Difference which corresponds to the difference in leaf temperature under water-deficit and well-watered conditions. The Crop Water Stress Index of Idso et al. (1981) and Jackson et al. (1981) is defined as the difference between air and canopy temperature (T_a_ and T_c_, respectively), normalized for the evaporative demand as determined by means of a lower limit LL (the case of a canopy transpiring at its potential rate) and an upper limit UL (a non-transpiring canopy):

(1) $$\begin{array}{l} \text{CWSI}=[\left({\text{T}}_{\text{c}}-{\text{T}}_{\text{a}}\right)-{\left({\text{T}}_{\text{c}}-{\text{T}}_{\text{a}}\right)}_{\text{LL}}]/[{\left({\text{T}}_{\text{c}}-{\text{T}}_{\text{a}}\right)}_{\text{UL}}\\ \;-{\left({\text{T}}_{\text{c}}-{\text{T}}_{\text{a}}\right)}_{\text{LL}}] \end{array}$$

The CWSI has to be calculated under clear sky conditions. It proved capable of predicting stress in plants 1–2 days before visual detection (Kacira et al., 2002). There are several methodological difficulties associated with the CWSI, including a high sensitivity to windy conditions. Other available indexes are the Temperature–Vegetation Dryness Index of Sandholt et al. (2002) or the Temperature Vegetation Index of Prihodko and Goward (1997). Generally, it can be said that, despite the progress of techniques and concepts, all these real-time, model-based indexes, for all the advantages they provide, are still lacking accuracy and require careful parameterization.

## Mesophyll conductance (g_m_)

Mesophyll conductance determines CO_2_ supply from sub-stomatal cavities to carboxylation sites. g_m_ has anatomical and physical characteristics, including CO_2_ solubility, the distribution of chloroplasts, the surface of chloroplasts exposed to the intercellular air space, surface area of intercellular spaces, walls and cytosol, and dimensions of the intercellular spaces which change as tissues and cells shrink with WD (Lawlor and Tezara, 2009; Tomas et al., 2013). The conductance through the liquid phase is generally believed to be the most limiting factor for CO_2_ diffusion in the mesophyll for many species (Flexas et al., 2012). g_m_ can change rapidly and independently of leaf anatomy, for instance it can decrease as a consequence of soil WD (Warren, 2008), supporting the view that g_m_ is also biochemical in nature. g_m_ depends on carbonic anhydrase activity, which facilitates CO_2_ transfer to Rubisco active sites, and has a metabolic component associated with aquaporins, which may act as CO_2_ channels (Mori et al., 2014). Of course, g_m_ can also decrease as a long-term response to WD (Gu et al., 2012; Han et al., 2016).

For years the importance of g_m_ has been underestimated in ecological and agronomical studies. Nowadays the quantitative importance of g_m_ in the control of photosynthesis has been well established but there are still ongoing controversies about estimation techniques. g_m_ can be estimated from joint measurements of gas exchange and chlorophyll fluorescence (Table 3), a common feature of the portable systems available on the market, using the constant electron transport rate (J) method (Bongi and Loreto, 1989; Harley et al., 1992), or the variable J method (Di Marco et al., 1990; Harley et al., 1992). g_m_ can also be estimated by the carbon isotope method (Evans et al., 1986; von Caemmerer and Evans, 1991; von Caemmerer et al., 2014), and by the so-called A-C_i_ curves fitting methods (Dubois et al., 2007; Sun et al., 2014; Sharkey, 2016). Important methodological difficulties are associated with evaluations of g_m_ (for a review see notably Warren and Dreyer, 2006; Pons et al., 2009; Tholen et al., 2012). There are all the more important that some assumptions associated with g_m_ estimation in current A-C_i_ curve-fitting methods introduce biases in fitting other model parameters. In spite of these difficulties and of debates (Warren, 2006; Warren and Dreyer, 2006; Lawlor and Tezara, 2009; Buckley and Warren, 2014), g_m_ has been going on fuelling a lot of interest among researchers during the last decade. Recently, Moualeu-Ngangue et al. (2017) presented a new method to fit A-C_i_ and Φ_PSII_-C_i_ curves simultaneously. Φ_PSII_ represents the quantum efficiency of photosystem II (PSII) in μmol electrons/μmol photons absorbed by PSII (Genty et al., 1989; Bilger et al., 1995). The newly described method of Moualeu-Ngangue et al. (2017), using the multiple phase flash approach for Φ_PSII_ (Loriaux et al., 2013), allows the estimation of the g_m_ dependence on C_i_.

## Metabolic vs. diffusional limitations to A_net_-evaluation of photosynthetic capacity

**Figure 2:**
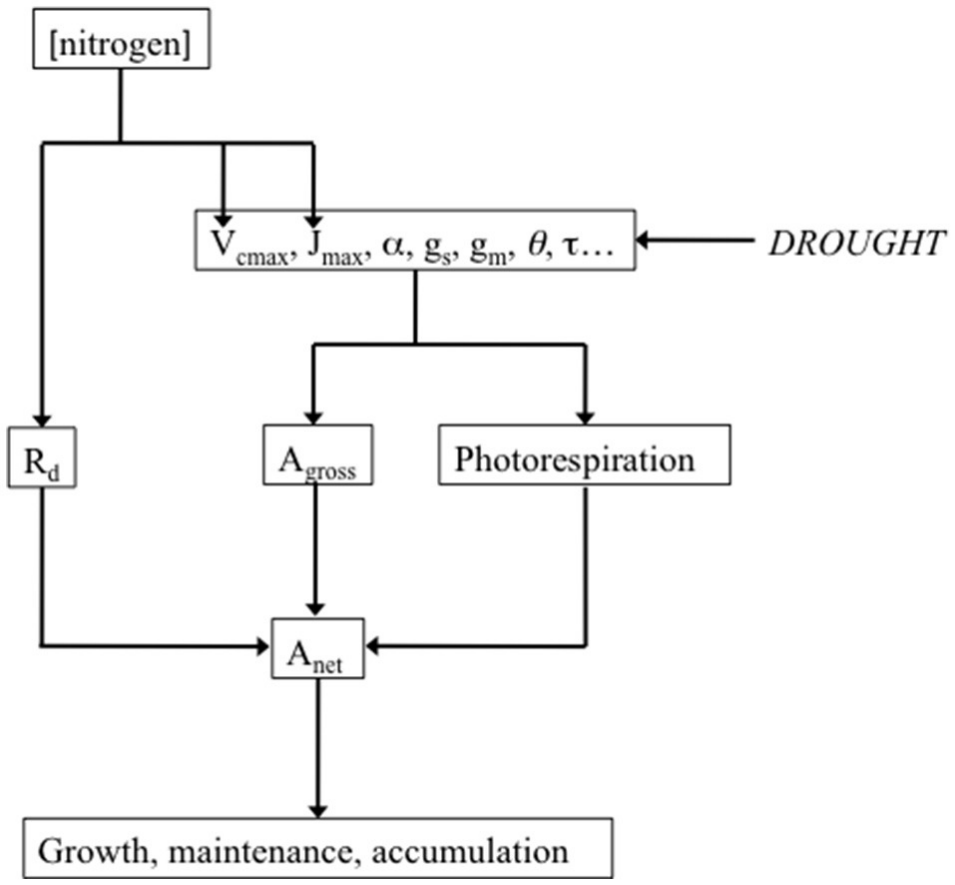
Drought potential impact on the major parameters of the biochemical model of leaf photosynthesis, and their link with net photosynthesis (A_net_). Drought potentially decreases the maximum carboxylation rate (V_cmax_), the light-saturated rate of electron transport (J_max_), the quantum efficiency of photosystem II (α), stomatal conductance to CO_2_ (g_s_), mesophyll conductance (g_m_), leaf absorbance (θ), the specificity factor of Rubisco (τ). All these parameters determine gross photosynthesis (A_gross_) and photorespiration, which, in addition to mitochondrial respiration (R_d_), in turn determine A_net_. Measuring and analyzing all these parameters can help understanding how drought impacts growth through A_net_. The influence of nitrogen on the determinants of photosynthetic capacity was represented as a reminder. Leaf nitrogen content expressed either on a leaf area (N_a_) or on a dry matter (N_m_) basis is generally well correlated with photosynthetic capacity (Field and Mooney, 1983; Evans, 1989; Kellomäki and Wang, 1997; Walcroft et al., 1997; Urban et al., 2003; Urban and Léchaudel, 2005; Kattge et al., 2009).

A decrease in A_net_ must not systematically be interpreted as a consequence of a drought-associated decrease in diffusional limitations of CO_2_ supply to carboxylation sites, i.e., a decrease in g_s_ or in the anatomical and physical components of g_m_. Indeed, A_net_ may also decrease as a consequence of metabolic limitations. An easy method to test the hypothesis of A_net_ limitation not associated to reduction in CO_2_ diffusion consists in using a high concentration of CO_2_ (Lawlor and Cornic, 2002). If the drought-associated decrease in A_net_ persists in such conditions, this will be considered as proof for the existence of non-diffusive limitations of photosynthesis. One common way of addressing this issue consists in measuring the maximal rate of net photosynthesis in conditions of non-limiting light and CO_2_ (A_max_). A non-diffusive decrease in A_max_ can generally be attributed to a decrease in one or more of the major components of photosynthetic capacity, namely V_cmax_, J_max_ and TPU (Figure 2), the maximum carboxylation rate, the light-saturated rate of electron transport and triose-phosphate utilization, respectively (Farquhar et al., 1980, 2001; Harley P. C. et al., 1992). V_cmax_ is related to Rubisco amount and activity, J_max_ represents the limitation to photosynthesis imposed by RuBP regeneration capacity, and TPU the limitation to photosynthesis imposed by triose-P utilization for starch and sucrose synthesis (Sharkey et al., 1986; Yang et al., 2016). The impact of WD on the amount and activity of Rubisco has been studied extensively. For Parry et al. (2002) drought can result can result in Rubisco deactivation. Lawlor and Tezara (2009) found that Rubisco activity is not very well correlated to decreases in A_net_. They consider that only severe WD can impact the content in Rubisco whereas Rubisco activity relates mainly on ATP status. There are numerous studies showing the impact of drought on J_max_. For instance, Martin-StPaul et al. (2012), studying three population of *Quercus ilex* in different sites, observed steeper declines of J_max_ as predawn leaf water potential declined in the wettest site compared with the drier sites (Flexas et al., 2004). discussed the impact of WD on sucrose phosphate synthase (SPS). SPS activity decreases as g_s_ decreases and would translate into a decrease in TPU. Damour et al. (2008) observed that photosynthetic capacity of leaves of lychee trees submitted to long-term drought decreases reversibly as a consequence reduced growth, sink activity, translocation and phloem loading.

V_cmax_, J_max_ and TPU are commonly calculated using the A-C_i_ curves (Table 3; von Caemmerer and Farquhar, 1981; Sharkey et al., 2007). Several assumptions behind the model underlying the A-C_i_ curves technique have been questioned and optimizing fits has been an important objective for the last years (Ethier and Livingston, 2004; Dubois et al., 2007; Sharkey et al., 2007; Gu et al., 2014; Duursma, 2015; Bellasio et al., 2016; Moualeu-Ngangue et al., 2017). Recently, Buckley and Diaz-Espejo (2015) stressed that there are methodological difficulties associated with J-Q submodels of photosynthesis, which result in underestimating J_max_ values. Alternative methods consist in exploiting light response curves or in incorporating the J-Q submodel directly into the photosynthesis model during the fitting process. Also, obtaining A-C_i_ curves is a time-consuming process because the leaf and gas exchange system is allowed to reach a steady state at each new applied [CO_2_]. Following ideas of Davis et al. (1987) and observations of McDermitt et al. (1989), Laisk and Oja (1998), and Stinziano et al. (2017) developed a novel A-C_i_ response technique, utilizing non-steady state measurements of gas exchange. Exploiting the capacity of the latest leaf gas exchange measurements systems to provide rapid control and measurement of step-wise changes in reference and sample [CO_2_], they showed that it is possible to reduce to less than 5 min the time necessary to determine A-C_i_ responses.

In addition to the A-C_i_ curve method, J_max_ can be calculated from measurements of ChlF following Smith (1937) and Harley P. C. et al. (1992). Urban et al. (2008) proposed to derive the initial quantum efficiency of PSII (α) and J_max_ from Φ_PSII_-Q curves (Table 3). So far, ChlF parameters derived from the analysis of OJIP/OKJIP transients have not been exploited to estimate photosynthetic capacity, and more specifically J_max_. In that prospect, it would certainly be interesting to evaluate the total number of electrons transferred into the photosynthetic electron transport chain (N), assuming that there is a strict proportionality between N and S_m_ (Stirbet, 2011), where S_m_ represents the normalized area of the ChlF induction curve. The high time resolution fluorimeters that can be purchased are either associated to portable leaf gas exchange measurement systems, like in the LI-6800, as stand-alone non modulated devices (like the Pocket PEA and the Handy PEA of Hansatech), or as stand-alone modulated devices such as the PAM-2500 of Walz or the PAR-FluorPEN FP 100-MAX of Photon Systems Instruments.

## Light absorption by leaves

Theoretically the capacity of the photosynthetic machinery to process CO_2_ is determined firstly by its capacity to capture light and to use absorbed energy by PSII (J^ABS^).

(2) $$\begin{array}{l} {\text{J}}^{\text{ABS}}={\text{Q}}^{*}\theta^{*}0.5 \end{array}$$

where J^ABS^ represents the rate of photon absorption by PSII antennae, Q the incident photosynthetically active quantum flux in μmol photons m^−2^ s^−1^ and θ the leaf absorbance. It is generally accepted that 50% of Q is absorbed by PSII and 50% by PSI. Massantini et al. (1990) observed a decrease in θ of water-stressed *Amaranthus* leaves. A decrease in θ would indeed help leaves to better cope with WD by reducing the amount of energy absorbed by photosystems and therefore the associated risk of photooxidative stress. There are few references about the effect of WD on θ and all of them are not confirming that WD results in a substantial decrease in θ (Osuna et al., 2015).

θ may be estimated from the formula: 1–absorbance of red light/absorbance of near infra-red light. Alternatively, θ can be evaluated exploiting correlations with leaf chlorophyll content (Table 3; Bauerle et al., 2004; Urban et al., 2008). One of the most popular instruments is the Chlorophyll meter SPAD 502® (Konica/Minolta, Osaka, Japan), which estimates leaf chlorophyll content based on the ratio of leaf transmittance between a chlorophyll non-absorbing wavelength and an absorbing one. Two other chlorophyll meters provide similarly precise and accurate measurements with different wavelength ratios. CCM-200® from Opti-Sciences Inc. (Hudson, USA) uses an equivalent transmittance ratio (653 and 931 nm) and Dualex 4® from Force-A (Orsay, France) uses a ChlF ratio (excited at 375 and 650 nm) (Cerovic et al., 2012). At sub-meter scale, an average chlorophyll content can also be estimated using the FIELDSCOUT CM-1000® (Spectrum Technologies Inc., Plainfield, USA).

Leaf light avoidance movements probably play an important role in light absorption reduction, notably in the short term. They could be monitored using imaging techniques. Clearly there is ample room for future developments in that direction.

## Efficiency of light conversion into photosynthetic electron transport-photoinhibition

The efficiency of absorbed light conversion, Φ_PSII_, determines, in addition to the amount of absorbed light, J^ABS^, the photosynthetic electron flux, J_T_ (alias J or ETR).

(3) $$\begin{array}{l} {\text{J}}_{\text{T}}=\Phi_{\text{PSII}}*{\text{J}}^{\text{ABS}} \end{array}$$

(4) $$\begin{array}{l} \Phi_{\text{PSII}}={\text{F}}_{\text{v}}^{\prime }/{\text{F}}_{\text{m}}^{\prime *}{\text{q}}_{\text{p}} \end{array}$$

where ${\text{F}}_{\text{v}}^{\prime }/{\text{F}}_{\text{m}}^{\prime }$ represents the quantum efficiency of so-called “open” (oxidized) PSII reaction centers and q_P_, photochemical quenching, the proportion of open PSII centers (Schreiber et al., 1986; Maxwell and Johnson, 2000).

(5) $$\begin{array}{l} {\text{F}}_{\text{v}}\prime ={\text{F}}_{\text{m}}\prime -{\text{F}}_{0}\prime \end{array}$$

where ${\text{F}}_{\text{m}}^{\prime }$ and ${\text{F}}_{0}^{\prime }$ represent the maximum value of ChlF under saturating illumination and the minimal ChlF, respectively, of light-adapted leaves.

${\text{F}}_{\text{v}}^{\prime }/{\text{F}}_{\text{m}}^{\prime }$ is correlated with the maximum quantum yield of primary PSII photochemistry, F_v_/F_m_, and with α (Urban and Alphonsout, 2007).

(6) $$\begin{array}{l} {\text{F}}_{\text{v}}={\text{F}}_{\text{m}}-{\text{F}}_{0} \end{array}$$

where F_m_ represents the maximum value of ChlF under saturating illumination, and F_0_, the initial (minimal) value of chlorophyll fluorescence, the level of fluorescence emission when all the primary quinone acceptors (Q_A_) are in the oxidized state, which is generally measured on dark adapted samples (Björkman and Demmig, 1987; Maxwell and Johnson, 2000; Roháçek, 2002). From a theoretical point of view, it is important to be aware that one of the major assumptions behind the interpretation of the fluorescence rise from minimal to maximal ChlF, including OJIP transients analysis, is that variable fluorescence is determined by the redox state of Q_A_, the first quinone acceptor of PSII, as originally proposed by Duysens and Sweers (1963). See Schansker et al. (2014) for a discussion about this hypothesis. From a practical point of view what is important is to ensure that both minimal and maximal ChlF are correctly measured. This is also true for OJIP transient analysis since they depend on normalizations that are very sensitive to the accuracy of the determination of F_0_ and F_m_ values (Kalaji et al., 2014). For useful considerations about dark adaptation, particularly in field trials (see also Kalaji et al., 2014).

The F_v_/F_m_ values average approximatively 0.83–0.84 in most C3 plants (Björkman and Demmig, 1987; Pfündel, 1998). Even though F_v_/F_m_ is arguably one of the most commonly used parameters derived from measurements of ChlF to assess plant stress, notably photoinhibition, i.e., photosynthesis reduction by excess of light, it remains generally unaffected by moderate drought (Genty et al., 1987; Tezara et al., 1999; Christen et al., 2007; Oukarroum et al., 2007). More severe WD may decrease F_v_/F_m_ values but, while substantial decreases in F_v_/F_m_ are indeed indicators of photo-damage, small decreases can be interpreted in terms of photo-protection (Adams et al., 2006). Similarly, a relatively moderate ${\text{F}}_{\text{v}}^{\prime }/{\text{F}}_{\text{m}}^{\prime }$-associated decrease in Φ_PSII_ may be interpreted as reduced risk of photo-oxidative stress. Even damage to D1 protein under WD, which indeed translates into lower values of q_P_ and F_v_/F_m_ (Giardi et al., 1996), can be seen as “positive photo-inhibition” since damaged D1 proteins are rapidly degraded and replaced.

In addition to the fluorimeters build in most recent portable gaz exchange measurement systems, the user can use dedicated modulated fluorimeter such as the FMS2 by Hansatech instruments (King's Lynn, UK), the Mini-PAM II by Walz, the OS5+ by Opti-Sciences, or the FluorPen FP 100-MAX of Photo Systems Instruments (Drasov, Czech Republic).

## Rerouting of electron fluxes (Figure 3)

Light reactions of photosynthesis convert the solar energy flux into chemical energy in the form of NADPH and ATP, which are needed for CO_2_ assimilation. In the case of drought, the photosynthetic electron transport rate can be reallocated from photosynthesis to photorespiration (Noctor et al., 2002; Galmès et al., 2007). In cotton it was observed that photorespiration increases as a consequence of drought (Cornic and Fresneau, 2002; Ennahli and Earl, 2005; Massacci et al., 2008; Chastain et al., 2014) but decreases have also been observed (Zhang et al., 2011). The glycolate oxidase and the Mehler peroxidase reactions respectively lead to the production of substantial amounts of H_2_O_2_ (a lesser evil than ^1^O_2_ and ${\text{O}}_{2}^{.-}$), either in peroxisomes or chloroplasts (Smirnoff, 1993; Noctor et al., 2002). Catalase, alongside several other enzymes and enzymatic systems, will then eliminate H_2_O_2_.

**Figure 3:**
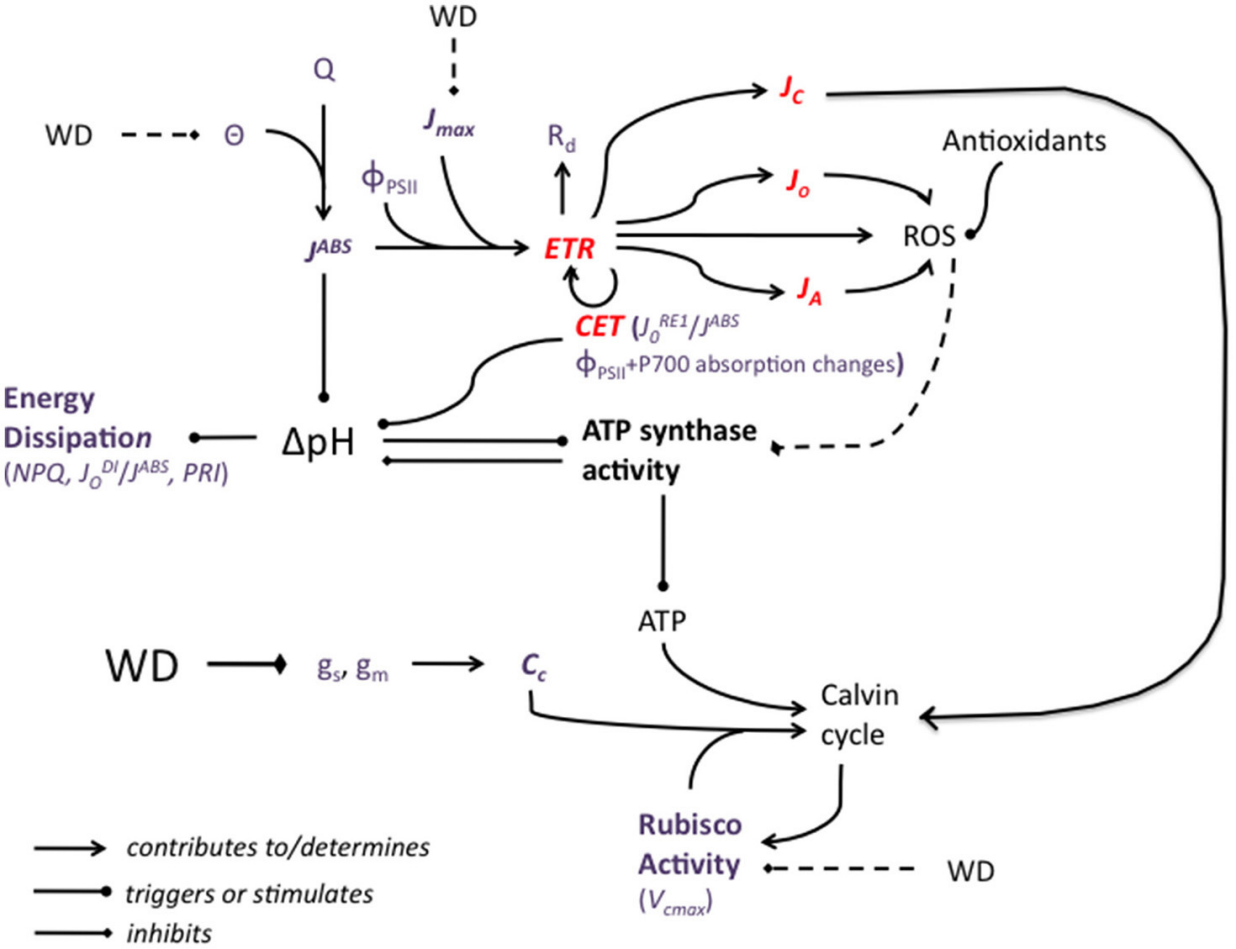
A simplified representation of the potential impact of water deficit (WD) on the major components of the photosynthetic machinery. WD decreases stomatal and mesophyll conductance, g_s_ and g_m_, leading to a decrease in the CO_2_ concentration at the carboxylation sites, C_c_. In conditions of high light, the slowing down of the Calvin cycle creates an energy imbalance and electron fluxes (ETR: electron transport rate) are rerouted from NADP^+^ reduction to photorespiration, to alternative electron sinks, to mitochondrial respiration, R_d_, and to the cyclic electron transport (CET). J_C_, J_O_, and J_A_ are the electron fluxes for carboxylation, oxygenation and alternative sinks, respectively. CET activity can be evaluated by measuring both Φ_PSII_ and P700- dependent absorption changes at 820 nm relative to 870 nm. ${\text{J}}_{0}^{\text{RE}1}$/J^ABS^ could also be used as an indicator of CET activity. Reactive oxygen species (ROS) may also be synthetized and they are not necessarily fully eliminated by ROS-scavenging molecules and processes. ROS have been hypothesized to damage ATP synthase, decreasing ATP production, which contributes again to slowing down the Calvin cycle. WD may impact negatively Rubisco activity (as assessed by the maximal carboxylation rate, V_cmax_) but a WD-associated decrease in V_cmax_ is more likely a consequence than a cause of the slowing down of the Calvin Cycle. Besides high light (Q) conditions, the cyclic electron transfert (CET), contributes to the trans-thylakoid H^+^ gradient, ΔpH, which drives ATP synthesis. ATP synthesis, by consuming protons, acts in the opposite direction. High ΔpH triggers excess absorbed energy (J^ABS^) dissipation processes, which can be evaluated by measuring non-photochemical quenching (NPQ), the ratio of dissipated on absorbed energy fluxes, ${\text{J}}_{\text{o}}^{\text{DI}}$/J^ABS^, or the photochemical reflectance index (PRI). The potential effect of WD on leaf absorbance (θ) and therefore J^ABS^ was represented as well as the effect of WD on the maximum rate of photosynthetic electron transport (J_max_). The effects of sucrose synthesis and phloem loading are not represented. Weak or controversial effects are represented by broken lines. Red characters and lines correspond to electron fluxes. Violet characters correspond to parameters that can be measured or calculated.

See Busch (2013) for a review of the existing methods for evaluating photorespiration. Both J_C_ and J_O_, the electron fluxes for carboxylation and for oxygenation, respectively, can be calculated using concomitant measurements of A_net_ and Φ_PSII_, using portable gas exchange + ChlF measurement systems, followed by measurements of R_d_ (Valentini et al., 1995). Prior calibration of Φ_PSII_ at 1–2% O_2_ must however be done (Genty et al., 1989). It is also in theory required to determine R_d_, the rate of mitochondrial respiration in light, and θ. The calibration procedure is time-consuming but can then be exploited to effect routine measurements on adequate plant material. The procedure can also be exploited to evaluate the electron flow to so-called alternative sinks, J_A_ (see Urban et al., 2008 for an example of field application of these methods). R_d_ plays a key-role in the photosynthetic carbon metabolism of leaves experiencing WD (Atkin and Macherel, 2009; Lawlor and Tezara, 2009), and also because it is an essential component of many models (J_C_, J_O_, J_A_, g_m_, τ…). By suppressing the light source, after equilibration, it is possible to easily measure R_n_, the rate of mitochondrial respiration in the absence of light. R_n_ is not equal to R_d_. There are however techniques to derive R_d_ from R_n_ following the methods of Kok (1948) or Laisk (1977). The latter has been widely exploited (Brooks and Farquhar, 1985; von Caemmerer et al., 1994; Peisker and Apel, 2001; Priault et al., 2006; Flexas et al., 2007; Urban et al., 2008). A method based on simultaneous measurements of ChlF and gas exchange (see below) has been proposed by Yin et al. (2009) and evaluated Yin et al. (2011). This method is valid for both C_3_ and C_4_ plants. More recently, the new method of Moualeu-Ngangue et al. (2017) which replaces g_m_ by the fraction of incoming photosynthetic photons harvested by PSII, was found to improve estimation of all major parameters derived from A-C_i_ curves analysis, including R_d_.

In oxygenic photosynthesis, the production ratio of ATP/NADPH by linear electron transport is about 1.29 whereas the ratio required by the Calvin cycle is 1.5 (Allen, 2002). In C_3_ plants, photorespiration increases the ratio up to 1.67 (Shikanai and Yamamoto, 2017). To satisfy the ATP/NADPH production ratio, supplementary mechanisms for ATP synthesis are needed. In cyclic electron transport (CET), electrons are transferred from ferredoxin to the plastoquinone pool, generating a trans-thylakoid H^+^ gradient via the Q cycle of Cyt *b*_6_*f* complex, without net production of NADPH (Yamori and Shikanai, 2016). The trans-thylakoid H^+^ gradient (ΔpH) is a major component of the proton motive force that contributes to ATP synthesis. The ΔpH also down-regulates photosynthetic electron transport by downregulating Cyt *b*_6_*f* complex activity and by evacuating absorbed light energy in excess under the form of heat from PSII antennae (Shikanai and Yamamoto, 2017). Apart from adjusting the ATP/NADPH ratio, the cyclic electron transfert (CET) participates in the development of non-photochemical quenching, NPQ (Niyogi, 2000), therefore affording protection against photooxidative stress (Martin et al., 2004). Besides, electrons from PSI which do not follow the linear electron transport route or the CET route are transferred to O_2_ to generate superoxide and other reactive oxygen species (ROS) that are normally scavenged by the water-water cycle. The water-water cycle consumes also reducing equivalents generated by PSI, ferredoxin, and NADPH. Besides the water-water cycle, nitrate reduction at PS I could also play an important role as an alternative electron sink (Bota et al., 2004). Chlororespiration is thought to participate in the regulation of CET activity by reducing plastoquinones (Rumeau et al., 2007). Shikanai and Yamamoto (2017) also formulated the hypothesis that CET activity could be influenced by electron transfer to the NADH dehydrogenase-like (NDH) complex by chlororespiration. The NDH complex was found to represent another pathway of PSI cyclic electron transfer in angiosperms.

It is possible to assess CET activity by measuring both Φ_PSII_ and P700- dependent absorption changes at 820 nm relative to 870 nm (Harbinson and Foyer, 1991; Klughammer and Schreiber, 1994; Kotakis et al., 2006; Huang et al., 2010), which is made possible by devices like the Dual-PAM of Walz. Alternatively, the electron transport fluxes from Q_B_ to PSI acceptors, ${\text{J}}_{0}^{\text{RE}1}$, expressed either as quantum yields (/J_ABS_) or per reactive centers (/RC) has been suggested as an indicator of CET activity (Ripoll et al., 2016b). ${\text{J}}_{0}^{\text{RE}1}$/J_ABS_ and ${\text{J}}_{0}^{\text{RE}1}$/RC can be derived from analysis of OJIP transients.

## Dissipation of excess energy

Leaves of water-stressed plants are commonly facing conditions characterized by an imbalance between the quantity of light energy absorbed relative to their capacity to deal with it through photosynthesis, photorespiration, or even alternative electron routes. The primary mechanism by which they transfer the absorbed light energy in excess away from photosynthetic electron transport toward heat production is energy-dependent quenching, which depends in part on the xanthophyll cycle (Horton and Ruban, 2005; Baker, 2008; Mozzo et al., 2008; García-Plazaola et al., 2012). So called non-photochemical quenching (attenuation) of ChlF, NPQ, increases as a consequence of WD, whereas photochemical quenching decreases (Tezara et al., 1999). There are two possible ways to evaluate dissipation of excess energy, either by using a traditional modulated fluorimeter, or by using a high time resolution fluorimeter. The first one provides crucial information about the importance of heat dissipation relative to photochemistry for given light conditions; the second provides information that rather has to be put into perspective with other parameters to assess the global strategy of the plant under investigation (Ripoll et al., 2016b).

NPQ can be calculated as (F_m_ – ${\text{F}}_{\text{m}}^{\prime }$)/ ${\text{F}}_{\text{m}}^{\prime }$ from measurements of maximal fluorescence performed on dark- (F_m_) and then light-adapted (${\text{F}}_{\text{m}}^{\prime }$) leaves (Schreiber et al., 1986; Bilger and Björkman, 1990; Bilger et al., 1995; Maxwell and Johnson, 2000; Müller et al., 2001; Kramer et al., 2004), using a standard modulated fluorimeter. Alternatively, q_N_ can be calculated as (F_m_ – ${\text{F}}_{\text{m}}^{\prime }$)/(F_m_ – ${\text{F}}_{0}^{\prime }$) (Schreiber et al., 1986; Kooten and Snel, 1990). Note that ${\text{F}}_{0}^{\prime }$ may be calculated instead of measured on light-adapted leaves, according to Oxborough and Baker (1997). There are however discrepancies. Recently, Ruban (2016) proposed a novel approach for analyzing light tolerance in plants, exploiting the discrepancy between calculated and measured ${\text{F}}_{0}^{\prime }$. It would certainly be of interest to test this approach for drought conditions.

Y_NPQ_ and Y_NO_, the quantum yield of light-induced non-photochemical quenching of fluorescence (associated to ΔpH and the xanthophyll cycle), and the yield of non-light induced non-photochemical quenching of fluorescence, respectively, are also useful parameters than can be easily calculated (Kramer et al., 2004). Y_NO_ corresponds to non-regulated dissipation of excess energy and may be used as an indicator of the stress-associated risk of photo-damage.

The new generation of portable fluorimeters, which provide the high time resolution required for performing measurements of fast ChlF induction kinetics, can be considered to facilitate analysis of heat dissipation even more easily than modulated fluorimeters since no light adaptation is required any more. But then leaves must be dark-adapted. The dissipated energy flux expressed on a PSII reaction center basis, J^DI^/RC, can be calculated as J^DI^/RC = J^ABS^/RC – ${\text{J}}_{0}^{\text{TR}}$/RC, where J^ABS^/RC represents the average absorbed photon flux per PSII reaction center (or, alternatively, the apparent antenna size of an active PSII), and ${\text{J}}_{0}^{\text{TR}}$/RC the maximum trapped exciton flux per PSII. J^ABS^/RC is calculated as (M_0_/V_J_)/(F_v_/F_m_) with M_0_ the initial slope of the relative variable ChlF curve, and V_J_ the value of relative variable ChlF at 2 ms. ${\text{J}}_{0}^{\text{TR}}$/RC is calculated as M_0_/V_J_ (Stirbet, 2011). J^DI^ can also be expressed per excited cross section: ${\text{J}}_{0}^{\text{TR}}$/CS. 1/(J^ABS^/RC), often noted as RC/ABS, is the first of the three ingredients of the popular, composite Performance Index on an absorption basis (PI_ABS_) of Strasser (Strasser and Srivastava, 1995; Srivastava and Strasser, 1999; Strasser et al., 2004; Stirbet, 2011). In addition to RC/J^ABS^, PI_ABS_ encompasses F_v_/F_0_ = (F_v_/F_m_)/(1 – (F_v_/F_m_)), an indicator of trapping probability, and (1 – V_J_)/ V_J_, an indicator of the performance of conversion of excitation energy to photosynthetic electron transport. PI_ABS_ is considered as a much more sensitive and discriminating stress indicator than F_v_/F_m_ (see for instance Le, 2007), even though contradictory observations in response to WD have been reported (Ripoll et al., 2016b). Differences in J^DI^/RC are generally discussed along with other variations in energy and electron fluxes, namely variations in the electron transport fluxes from Q_A_ to Q_B_, ${\text{J}}_{0}^{\text{ET}2}$, and in ${\text{J}}_{0}^{\text{RE}1}$. When compared to PI_ABS_, ${\text{PI}}_{\text{ABS}}^{\text{TOT}}$ actually includes an additional parameter related to electron transport flux to PSI acceptors.

Alongside parameters derived from chlorophyll *a* fluorescence, the Photochemical Reflectance Index (PRI) of Gamon et al. (1992) may be used to evaluate the epoxidation rate of xanthophylls, which was observed to result in a major shift in reflectance at 531 nm compared to stable reflectance at either 515, 550, or 570 nm. Because xanthophyll cycle pigments adjust the energy distribution at the photosynthetic reaction center, the PRI can be considered as an indicator of photosynthetic light use efficiency (LUE) and of stress (Gamon et al., 1992). Even though the PRI is highly sensitive to light conditions, it has been found to be particularly useful for measuring vegetation health status at the canopy and field scale, prior to senescence. A normalized version of the PRI has been proposed by Zarco-Tejada et al. (2013) which allows for corrections for both canopy density and chlorophyll content variations. The PRI has already been used successfully as an indirect water stress indicator (Thenot et al., 2002; Peguero-Pina et al., 2008; Suárez et al., 2008, 2009, 2010). As portable commercial sensors measuring PRI and NDVI are now available, PRI time series becomes easy to acquire. At the short-term scale, PRI is a promising physiological indicator of stresses. However, PRI value is affected by tissue structural changes, chlorophyll content level and carotenoid/chlorophyll content ratios (Sims and Gamon, 2002; Wong and Gamon, 2015). Consequently, the relationships between light use efficiency (LUE) and PRI, between F_v_/F_m_ and PRI (Stylinski et al., 2002), and between ΔF/Fm′ and PRI (Gamon et al., 1997), are specific of plant species and of growing condition. By using PRI values of dark-adapted leaves (PRI_0_), which are highly correlated to chlorophyll content, saturating Q and soil moisture, it is possible to define PRI seasonal variations, and then to analyze short-term variations which are correlated to light interception and LUE (Hmimina et al., 2014, 2015). The occurrence of clouds affects directly and negatively PRI (Merlier et al., 2015). PRI variations are greater in sunlit upper leaves than in the shaded leaves found inside the canopy, reflecting a higher investment of the photoprotective xanthophyll cycle pigments (Gamon and Berry, 2012). Some caution should be observed when comparing PRI values among younger and mature leaves at a given time period, and when comparing PRI values at different seasons. Pigment content analysis in contrasted conditions is recommended for relevant interpretation of PRI variations. The correlation between PRI and F_v_/F_m_ is no longer verified when senescence starts. During extreme drought, PRI can become decoupled from LUE, leading to overestimates of LUE (Gamon et al., 2001; Filella et al., 2004; Nakaji et al., 2006; Rahimzadeh-Bajgiran et al., 2012).

## Antioxidant metabolism

The antioxidant metabolism in plants encompasses enzymatic and non-enzymatic processes. It is known since long that there are both strongly influenced by WD (Reddy et al., 2004; Nakabayashi et al., 2014). To evaluate enzymatic processes, it is needed to measure the activities of antioxidant enzymes like superoxide dismutase and of enzymes of the antioxidant systems (Poiroux-Gonord et al., 2013). There are no non-destructive methods so far that can be used in the field to evaluate enzymatic activities. By contrast, there are field techniques for evaluating the content in non-enzymatic antioxidant molecules. [anthocyanins] and [flavonols] can be measured at least using *in vivo*, non-destructive measurements of ChlF based on the fluorescence excitation ratio method (Bilger et al., 1997; Agati et al., 2011). The method was developed for canopies (Ounis et al., 2001) and tested also on fruits (see for instance Betemps et al., 2012). The Dualex® and the Multiplex® systems that are used on leaves make use of a reference beam of red light (not absorbed by flavonols and anthocyanins) and one or more additional beams providing different excitation wavelengths. UV-A is strongly absorbed by flavonols whereas green light is strongly absorbed by anthocyanins (Cerovic et al., 2002, 2012; Goulas et al., 2004; Cartelat et al., 2005; Bürling et al., 2013). Diodes for detecting fluorescence emission at 590, 685, and 735 nm allow corrections for differences in chlorophyll content in leaves since the red/far red fluorescence ratio is related to chlorophyll concentration (Hák et al., 1990; Lichtenthaler et al., 1990; Buschmann et al., 2001; Buschmann, 2007; Gameiro et al., 2016). Apparently, using either a blue or a red reference light beam to make measurements on green leaves was not found to influence results (Cerovic et al., 2002, 2012; Goulas et al., 2004; Cartelat et al., 2005; Pfündel et al., 2007; Bürling et al., 2013). It must be noted that the specific modulated fluorimeters that are used to measure [anthocyanins] and [flavonols] in leaves can be easily operated in the field with the added bonus of little influence of current climatic parameters. It must however be kept in mind that the no units data provided must be corrected to be expressed on dry matter basis.

## Damage indicators

At some point, stress may not simply trigger acclimation mechanisms but also result in various damages (Figure 1). Most damage-related parameters that can be measured in the field derive from ChlF measurements or are indicators of leaf chlorophyll content. We propose to consider here five ChlF parameters: F_o_, the relative variable ChlF at 300 μs, NPQ, the normalized area of the fluorescence induction curve, and, tentatively, the probability of connectivity.

An increase in F_0_ may be caused by the release of free chlorophyll from protein-pigment complexes, which results in blocked energy transfer to the PSII traps (Armond et al., 1978, 1980; Sundby et al., 1986). An increase in F_0_ may not be reflected in a decrease in F_v_/F_m_ when there is a concomitant decrease in F_m_. A decrease in F_m_ is a common occurrence in conditions of stress, since a decrease in F_m_ reflects sustained engagement of zeaxanthin in a state primed for energy dissipation, i.e., the stimulation of the photoprotective mechanism known as the xanthophyll cycle (Wingler et al., 2004).

Drought may cause damage to the oxygen-evolving center (OEC) coupled with PSII (Kawakami et al., 2009), besides of degradation of D1 protein (He et al., 1995; Giardi et al., 1996), leading to inactivation of the PSII reaction centers (RC) (Liu et al., 2006; Zlatev, 2009), which may eventually lead to ROS generation as well as photoinhibition and oxidative damage (Ashraf, 2009; Gill and Tuteja, 2010). Limitation/inactivation, possibly damage of the OEC may be observed and assessed through the increase in relative variable fluorescence at 300 μs (K-step), V_K_ (Srivastava et al., 1997), although such an increase may also be interpreted as a different functional antenna size (Yusuf et al., 2010). The V_K_/V_J_ ratio can also be used as a relative measurement of the functional antenna size (Yusuf et al., 2010) or of OEC inactivation/damage (Kalachanis and Manetas, 2010; see also Kotakis et al., 2014). V_J_ stand for relative variable fluorescence at 2 ms. A K-step occurs whenever the electron flow to the acceptor side exceeds the electron flow from the donor side. This leads to RC oxidation with a photosystem shift toward the ${\text{P}}_{680}^{+}$ form which is known to have a low fluorescence yield (Srivastava et al., 1997). Thus, OEC dissociation triggers the K-step, by inhibiting efficient electron donation to the RC (Strasser, 1997; De Ronde et al., 2004). The appearance of the K-band is associated with heat and drought stress. Christen et al. (2007) observed indeed an increase in F_K_ as a consequence of drought. Similarly, Oukarroum et al. (2007) observed that the K-band can be exploited to analyse responses to drought stress in barley cultivars.

It was hypothesized that the repair cycle for ATP synthase components is not as active as for D1 protein (Nishiyama et al., 2001). Mahler et al. (2007) observed that ^1^O_2_ damages result in a decrease in ATP hydrolysis and increased NPQ. Considering that ATP hydrolysis strongly correlates with ATP synthase activity, substantially increased NPQ may be an indicator of damage to ATP synthase.

S_m_ is the normalized area of the fluorescence induction curve. S_m_ is assumed to be proportional to the pool size of electron carriers (Yordanov et al., 2008). The plastoquinone pool may indeed decrease as a consequence of stress (Bishop, 1961; Shavit and Avron, 1963) but then probably only in case of severe stress. For example, Christen et al. (2007) observed that moderate drought did not affect S_m_ in grapevine.

The shape of the induction curve between 50 and 300 μs (so-called L-band) is influenced by the excitation energy transfer between PSII units, commonly denoted as connectivity (Strasser and Stirbet, 1998). A more hyperbolic transient is a reflection of an increase in the energetic connectivity and a decrease can be observed as a consequence of drought (Oukarroum et al., 2007). Therefore, p, the probability of connectivity, which can be derived according to the method described by Stirbet (2011), could be an indicator of damage.

As stress intensifies, chloroplasts will ultimately break down. A large proportion of nitrogen resources are tied up in leaves, mostly in chloroplasts, and these resources can be redistributed elsewhere (Lawlor, 1993). Decreases in leaf nitrogen or chlorophyll content are therefore ultimate indicators of severe stress. There is some evidence that WD may accelerate loss of leaf nitrogen and chlorophyll, and enhance senescence as it was observed in wheat (Yang et al., 2001). Recently, Okami et al. (2016) observed that the optimal vertical distribution of leaf nitrogen content expressed on leaf mass basis, N_m_, may be affected by drought in an *indica* cultivar of rice. But generally it takes severe stress before the structure, not purely the functioning, of the photosynthetic machinery is affected. Weak or very progressive long-term drought seems to impact only weakly, if at all, leaf nitrogen content (Sinclair et al., 2000; Damour et al., 2008).

Nitrogen content determination is time consuming but there are also indirect, fast and non-destructive methods derived from estimates of chlorophyll content (see above). It is however important to remember that the chlorophyll-nitrogen relationship depends on the growing season and on nitrogen content range (Evans, 1983). Also the influence of light intensity when using a chlorophyll meter must be taken into account since chloroplasts are known to rearrange themselves inside the cell in response to blue light intensity (Sakai et al., 2001; Kasahara et al., 2002). Parameters used in remote sensing, such as the ratio between ChlF at 735 and 700 nm, which is linearly proportional to chlorophyll content (Gitelson et al., 1999), can be used to evaluate leaf nitrogen at leaf or plant scale. Alternatively, leaf nitrogen content per unit leaf area, N_a_, can be estimated for instance from R_1075_/R_735_ reflectance ratios or, better still, from the ratio dR/dλ at 740 nm (Zhao et al., 2005). More recently, Vigneau et al. (2011) proposed to use hyperspectral imaging to assess N_m_ in wheat.

## Conclusion

Assessing water status and the physiological responses triggered by WD has been a major challenge in plant science for decades. This challenge has become even more important in the context of global change. Nothing less than our capacity to manage dwindling water resources and to ensure food security for the world population is at stakes here. Not surprisingly, we have been observing for several years an outburst of new concepts, innovative techniques and novel parameters. Obviously, parameters derived from ChlF measurements, either alone or combined with parameters derived from gas exchange techniques, will play an increasingly important role in analyzing the impact of WD on photosynthesis. Most of these parameters being easy to obtain in the field, it is our belief that they will be increasingly exploited to explore dimensions of the complexity of plants' responses to WD that have been neglected so far in agronomic studies notably. We may have a relatively precise vision of the short-term molecular response of a potted *Arabidopsis* plant, grown in the stable environment of a phytotron, when subjected to a brutal interruption of water supply; however we are far from being able to predict what happens in the field to plants of variable genetic background and developmental stages, submitted to periods of more or less severe drought, possibly interrupted by periods of recovery, while other environmental factors, including pests and pathogens, fluctuate and interact with them (Ripoll et al., 2016a).

## Author contributions

LB contributed specifically to the stomatal conductance and mesophyll conductance sections. He is also the author of all the parts of the text dealing with remote sensing techniques. JA contributed namely to the section about damage indicators. LU is the major contributor to all other sections.

### Conflict of interest statement

The authors declare that the research was conducted in the absence of any commercial or financial relationships that could be construed as a potential conflict of interest.
